# Comprehensive Analysis of Salmonella Species Antibiogram and Evolving Patterns in Empirical Therapy: Insights From Tertiary Care Hospitals in Peshawar, Pakistan

**DOI:** 10.7759/cureus.57110

**Published:** 2024-03-28

**Authors:** Rizwan Ullah, Aiysha Gul, Faiza Gul, Nida Gul, Suleman Khan, Khayam, Waqar Khan, Kashif Ali, Aman ullah, Irum Rehman

**Affiliations:** 1 Internal Medicine, Hayatabad Medical Complex Peshawar, Peshawar, PAK; 2 Gynecology, Mardan Medical Complex, Mardan, PAK; 3 Paediatrics, Lady Reading Hospital, Peshawer, PAK; 4 Medicine and Surgery, Khyber Medical College, Peshawer, PAK; 5 Internal Medicine, Combined Military Hospital, Peshawar, PAK; 6 Medicine and Surgery, Hayatabad Medical Complex Peshawar, Peshawar, PAK; 7 Internal Medicine, Lady Reading Hospital, Peshawar, PAK; 8 Emergency Department, Khalifa Gul Nawaz Teaching Hospital, Bannu, PAK; 9 Gastroenterology, Ayub Teaching Hospital, Abbottabad, PAK

**Keywords:** empirical therapy, antibiotic sensitivity, antibiotic resistance, salmonella species, pakistan peshawar, meropenem, amikacin, fosfomycin, enteric fever, antibiogram

## Abstract

Background

Typhoid fever presents a significant challenge in developing nations, exacerbated by the emergence of antibiotic-resistant strains due to widespread prevalence and overuse of antibiotics. This study seeks to assess the antibiogram profiles of Salmonella species isolated from blood cultures of patients hospitalized at two prominent tertiary care hospitals in Peshawar, Pakistan: Khyber Teaching Hospital (KTH) and Hayatabad Medical Complex (HMC). By examining these profiles, the research aims to provide valuable insights into the evolving landscape of antibiotic resistance in the context of typhoid fever management.

Materials and Methods

This retrospective cross-sectional study utilized data gathered from two hospitals in Peshawar, KTH and HMC. Cases of enteric fever were identified based on positive blood cultures for Salmonella species. The study encompasses demographic information, seasonal prevalence, and antibiogram profiles of 3,137 cases that were presented between 2017 and 2023.

Results

Among the total 3,137 cases, males accounted for the majority, comprising 63% (2,044 cases). Particularly notable was the clustering of cases among children and adolescents aged one to 24 years. The incidence peaked during the months of summer and spring, from April to September. In terms of Salmonella Typhi isolates, considerable resistance was noted against first-line antibiotics such as amoxicillin/clavulanate (80.1%), co-trimoxazole/trimethoprim-sulfamethoxazole (66.6%), and chloramphenicol (86.9%), as well as against ceftriaxone (79.7%) and ciprofloxacin (51.6%). Conversely, certain antibiotics displayed higher sensitivity patterns, including meropenem (97.8%), doripenem (99.5%), imipenem (97.7%), ertapenem (96.5%), polymyxin B (99.4%), colistin (98.1%), and tigecycline (97.3%). Despite a limited sample size of 214 specimens, fosfomycin demonstrated a remarkable sensitivity of 93.4%. Sensitivities of amikacin and gentamicin were 90.7% and 81.5%, respectively. However, the sensitivity of azithromycin was concerning, standing at 66.5%. The antibiogram pattern for Salmonella exhibited significant and drastic changes.

Conclusion

In conclusion, this study sheds light on a higher prevalence of typhoid fever among males, with a notable seasonal peak observed during the summer and spring months. The age group most affected spans from one to 24 years. Salmonella isolates displayed significant resistance to conventional first-line antibiotics, alongside ciprofloxacin and third-generation cephalosporins. Azithromycin exhibited lower sensitivity compared to amikacin, gentamicin, and fosfomycin. The research advocates for the empirical use of amikacin, gentamicin, fosfomycin, and meropenem in the treatment of typhoid fever in Pakistan. Urgent measures, including regular Salmonella antibiogram surveillance, antibiotic stewardship, public health education, and Salmonella vaccination programs, are deemed crucial for primary disease prevention.

## Introduction

Salmonella Typhi (S. Typhi), which causes typhoid fever, and Salmonella Paratyphi (S. Paratyphi), responsible for paratyphoid fever, are the culprits behind enteric fever, an intestinal tract illness. Individuals present with fever, malaise, constipation, and stomach pain. Globally, there are 5.4 million cases of paratyphoid fever and 21.6 million cases of typhoid fever each year, resulting in 250,000 deaths. Asia accounts for approximately 80% of cases and fatalities. In India, the incidence of enteric fever ranges from 102 to 2,219 per 100,000 people. Typhoid fever has a significant morbidity and mortality rate if left untreated. Typhoid fever is expected to have a 30% fatality rate if treatment is not received [1].

In Pakistan, enteric fever is a serious public health issue. In Karachi, children are estimated to have an annual incidence of S. Typhi and S. Paratyphi of 451/100,000 and 76/100,000, respectively [2]. One of the most dangerous side effects of typhoid fever is intestinal perforation. In addition to the above-listed conditions, typhoid fever can also cause pneumonia, meningitis, endocarditis, osteomyelitis, and arthritis. Typhoid fever symptoms might, therefore, be related to several organs, which frequently lead to misdiagnosis. A typhoid fever diagnosis requires the organism to be isolated from the blood culture [3].

In the past, co-trimoxazole/trimethoprim-sulfamethoxazole, ampicillin, and chloramphenicol were the first-choice antibiotics for treating enteric fever [4]. Multidrug-resistant [(MDR) resistant to all first-line antibiotics] Salmonella strains have been identified since the late 1980's. Fluoroquinolone is the recommended medication for MDR typhoid strains that are susceptible to quinolones (WHO recommendations 2003). Nonetheless, nalidixic acid-resistant bacteria with decreased susceptibility to fluoroquinolones have emerged in Nepal as a result of the extensive usage of fluoroquinolones [5]. It was discovered that over 60% of typhoid fever isolates in Kolkata and Karachi were resistant to nalidixic acid [6]. Third-generation cephalosporin use has increased as a result of this. Nonetheless, reports of third-generation Salmonella strains resistant to cephalosporins have come from India and Nepal. In Pakistan, there was a widespread drug-resistant (XDR) S. Typhi outbreak between 2016 and 2017 [7]. The fact that there are fewer and fewer treatment alternatives available to physicians, along with rising treatment prices, is quite concerning.

Since drug-resistant isolates of Salmonella have emerged, first-line antibiotics have become less popular and are rarely used to treat this illness. Remarkably, reports of Salmonella strains in India and Nepal that are responsive to first-line antibiotics have surfaced recently [5, 8]. It is encouraging that Salmonella strains responsive to first-line antibiotics are resurfacing, and this emphasizes the significance of ongoing antibiogram surveillance.

This study aims to identify the antibiogram profile of Salmonella species isolated from blood cultures of patients who present to KTH Peshawar and HMC, two large tertiary care hospitals in Khyber Pakhtunkhwa (KPK). The idea is to provide suitable empirical antibiotics for enteric fever, considering evolving antibiograms and rising drug resistance.

## Materials and methods

The retrospective cross-sectional study conducted between January 2017 and July 2023 at HMC and KTH in Peshawar, Pakistan, aimed to comprehensively evaluate the antibiogram profiles of Salmonella species isolated from patients with positive blood cultures indicative of enteric fever. Utilizing a non-probability consecutive sampling approach, a total of 3,137 positive cultures were included in the analysis, encompassing patients across various age groups, ranging from less than a year old to 84 years old.

The study focused on gathering demographic information, assessing seasonal prevalence trends, and analyzing antibiogram profiles to understand the patterns of antibiotic resistance and susceptibility. Antibiotic resistance and susceptibility were assessed using the World Health Organization's software, WHONET, and BACLINK version 2023, evaluating the following antibiotics: ampicillin, amoxicillin/clavulanic acid, piperacillin/tazobactam, cefoperazone, ceftazidime, ceftriaxone, cefotaxime, cefepime, cefixime, aztreonam, doripenem, ertapenem, imipenem, meropenem, amikacin, gentamicin, ciprofloxacin, levofloxacin, moxifloxacin, co-trimoxazole/trimethoprim-sulfamethoxazole, fosfomycin, colistin, polymyxin B, azithromycin, chloramphenicol, and tigecycline.

In defining multidrug resistance (MDR) and extensively drug-resistant (XDR) strains, the study categorized MDR as resistance to first-line antibiotics, namely cotrimoxazole, chloramphenicol, and amoxicillin. XDR strains were defined as exhibiting resistance not only to first-line antibiotics but also to ciprofloxacin and ceftriaxone, encompassing fluoroquinolones and third-generation cephalosporins.

However, the study encountered limitations, notably the lack of analysis based on specific Salmonella strains due to the records from the hospitals only indicating the presence of Salmonella species without specifying the particular strain. Ethical approval for the study was obtained from the Ethical Review Board (ERB) of both HMC and KTH in Peshawar. The ERB of HMC provided approval with the number 1736, while KTH provided approval with the number 347/DME/KMC.

This comprehensive methodology allowed for a detailed examination of the antibiogram profiles of Salmonella species, providing valuable insights into the antibiotic resistance landscape and informing strategies for the effective management of enteric fever in Pakistan.

## Results

**Figure 1:**
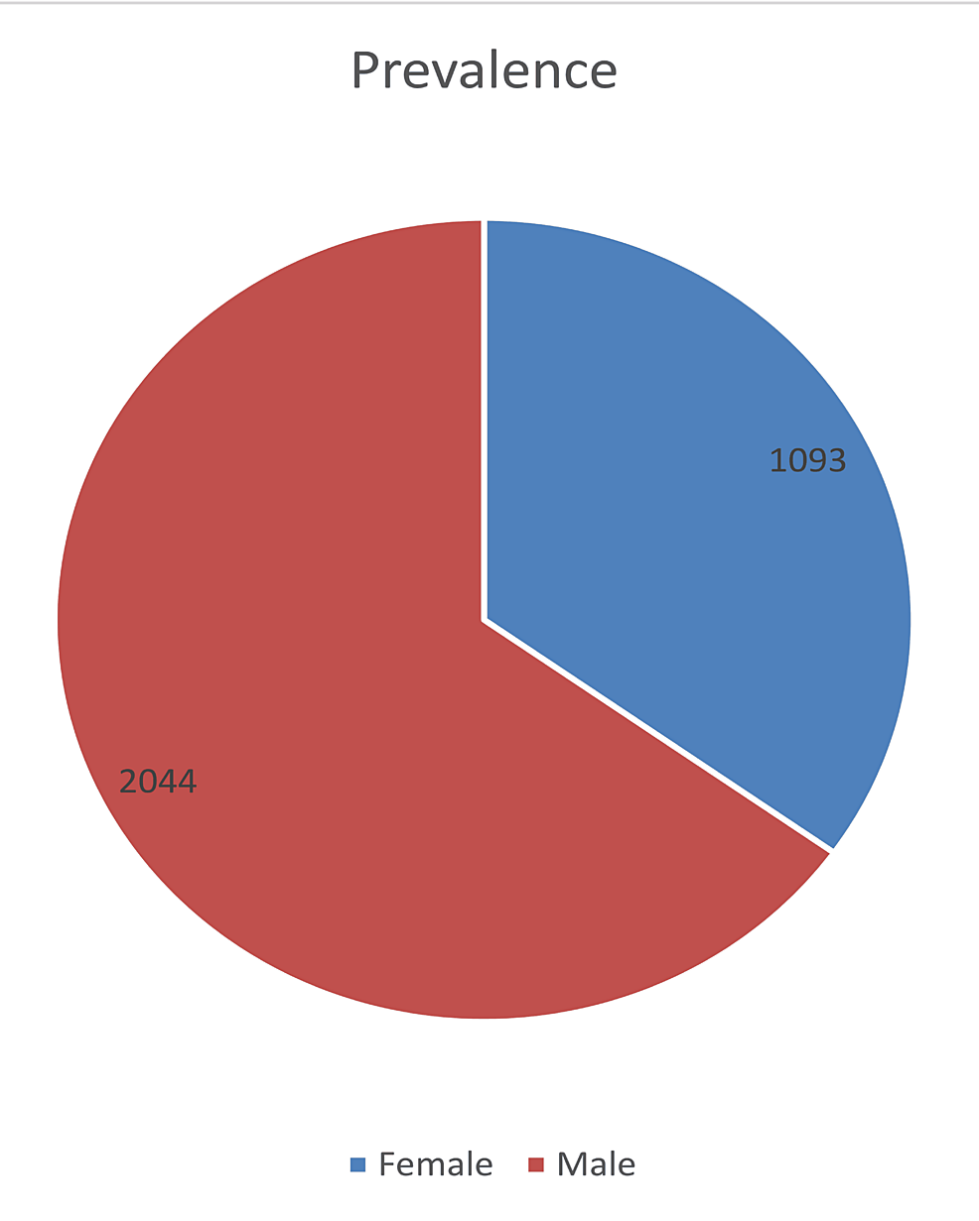
Gender-wise prevalence of typhoid fever

In this study, we analyzed a total sample size of 3,137 individuals, comprising 1,093 (35%) females and 2,044 (65%) males, as depicted in (Figure 1). 

**Figure 2:**
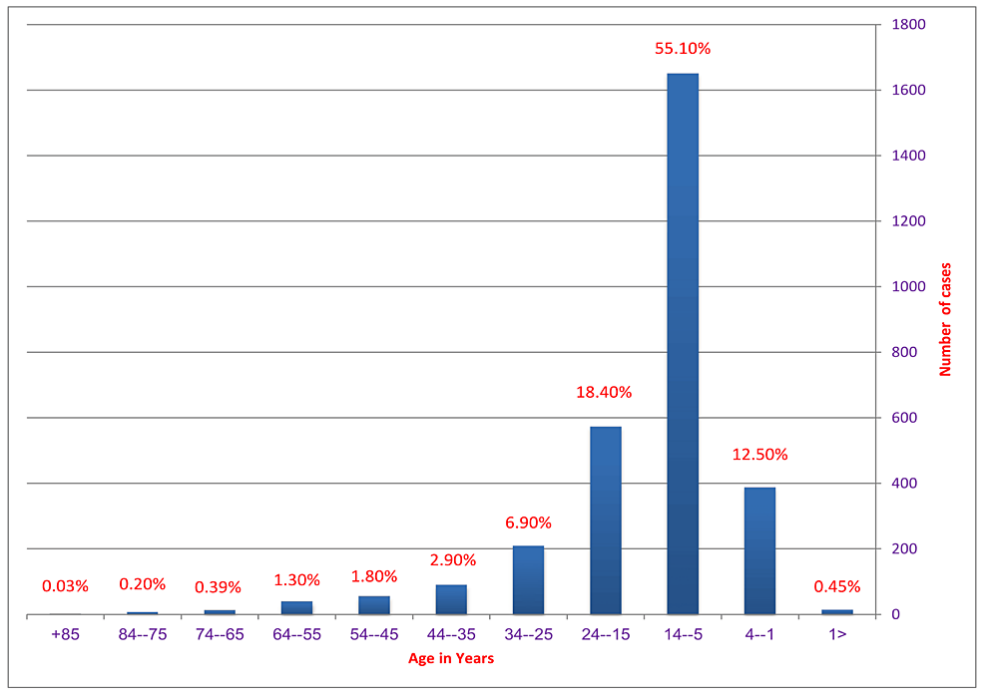
Age-wise prevalence. The highest prevalence seen was in age group 5-14 years age group (55.10%)

The most affected age group was five to 14 years old, accounting for 55% of the cases. Age-specific prevalence is illustrated in (Figure 2) and (Table 1). 

**Table 1:** Age-wise distribution of typhoid fever

Age	Number of positive cases	Percent (%)
<1	14	0.45%
1--4	394	12.50%
5--14	1,729	55.10%
15--24	577	18.40%
25--34	216	6.90%
35--44	90	2.90%
45--54	56	1.80%
55--64	40	1.30%
65--74	12	0.39%
75--84	7	0.20%
85+	1	0.03%

**Figure 3:**
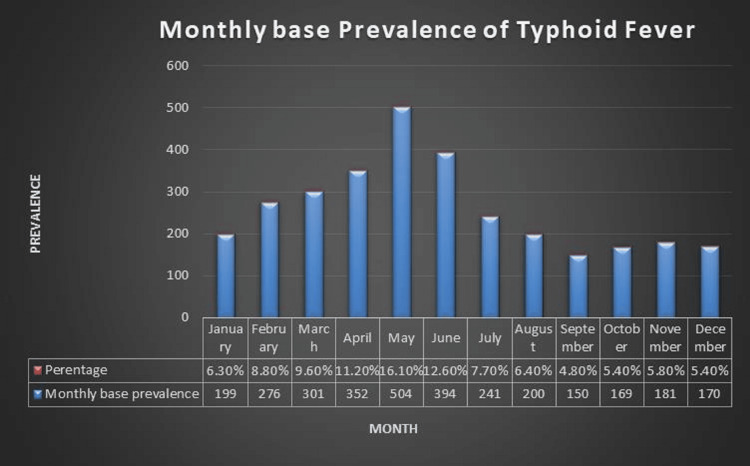
Monthly base prevalence of typhoid fever

The seasons with the highest number of cases were summer and springtime, with April (11.20%), May (16.10%), and June (12.60%) showing the highest incidence. These data are presented in (Figure 3) and (Table 2).

**Table 2:** Monthly base prevalence of typhoid fever

Percent (%)	Monthly base Prevalence	Month
6.30%	199	January
8.80%	276	February
9.60%	301	March
11.20%	352	April
16.10%	504	May
12.60%	394	June
7.70%	241	July
6.40%	200	August
4.80%	150	September
5.40%	169	October
5.80%	181	November
5.40%	170	December

**Figure 4:**
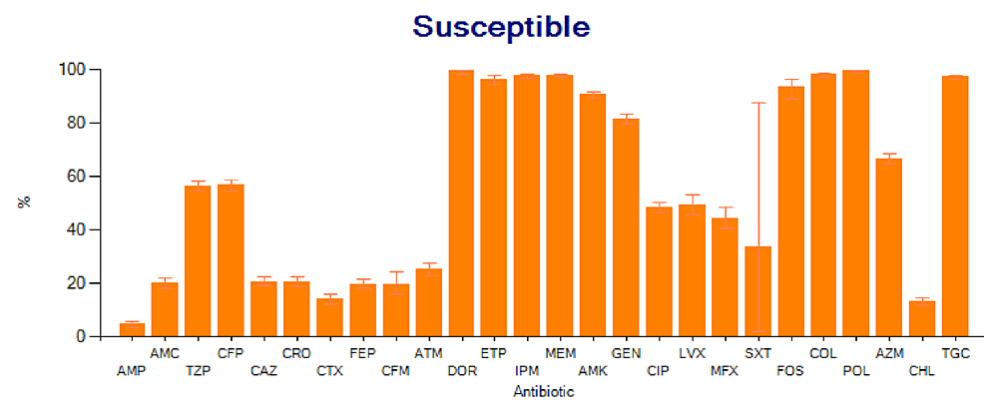
Salmonella antibiogram susceptibility pattern AMP: Ampicillin, AMC: Amoxicillin/Clavulanic acid, TZP: Piperacillin/tazobactum, CFP: Cefoperazone, CAZ: Ceftazidime, CRO: Ceftriaxone, CTX: Cefotaxime, FEP: Cefepime, CFM: Cefixime, ATM: Aztreonam, DOR: Doripenem, ETP: Ertapenem, IPM: Imipenem, MEM: Meropenem, AMK: Amikacin, GEN: Gentamicin, CIP: Ciprofloxacin, LVX: Levofloxacin, MFX: Moxifloxacin, SXT: Trimeth0prim/sulfamethoxazole, FOS: Fosfomycin, COL: Colistin, POL: Polymyxin B, AZM: Azithromycin, CHL: Chloramphenicol, TGC: Tigecycline.

**Figure 5:**
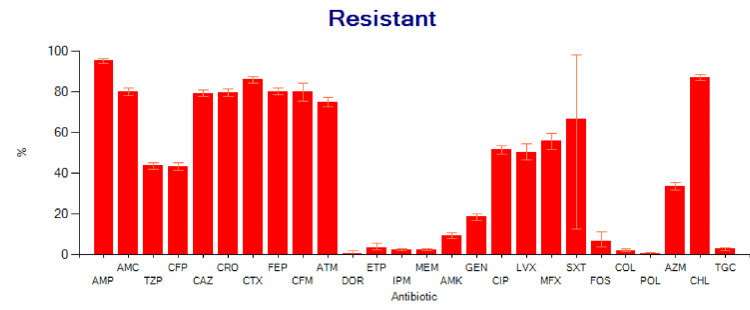
Salmonella antibiogram resistant pattern AMP: Ampicillin, AMC: Amoxicillin/Clavulanic acid, TZP: Piperacillin/tazobactum, CFP: Cefoperazone, CAZ: Ceftazidime, CRO: Ceftriaxone, CTX: Cefotaxime, FEP: Cefepime, CFM: Cefixime, ATM: Aztreonam, DOR: Doripenem, ETP: Ertapenem, IPM: Imipenem, MEM: Meropenem, AMK: Amikacin, GEN: Gentamicin, CIP: Ciprofloxacin, LVX: Levofloxacin, MFX: Moxifloxacin, SXT: Trimeth0prim/sulfamethoxazole, FOS: Fosfomycin, COL: Colistin, POL: Polymyxin B, AZM: Azithromycin, CHL: Chloramphenicol, TGC: Tigecycline.

The results of the antibiogram analysis reveal notable sensitivities among certain drugs: meropenem [97.8%, 95% confidence interval (CI) of 97.2-98.3], doripenem (99.5%, 95% CI of 98.1-99.9), imipenem (97.7%, 95% CI of 97.0-98.3), ertapenem (96.5%, 95% CI of 94.6-97.9), polymyxin B (99.4%, 95% CI of 98.9-99.8), colistin (98.1%, 95% CI of 97.3-98.8), and tigecycline (97.3%, 95% CI of 96.5-97.9). Although only 214 specimens were tested, fosfomycin shows intriguing sensitivity, with a percentage of 93.4% and a CI of 95% (89.0-96.2). Amikacin also exhibits high sensitivity, with a percentage of 90.7% and a 95% CI of 89.4-91.9. Gentamicin shows a sensitivity of 81.5% with a 95% CI of 79.8-83.2%. However, the sensitivity of azithromycin (66.5%) is concerning, with a 95% CI of 64.6-68.5%. Conversely, higher resistance is observed with third-generation cephalosporins, the penicillin group, chloramphenicol, and trimethoprim-sulfamethoxazole (co-trimoxazole). The below table presents these findings (Table 3), while Figures 4, 5 offer graphical representations of the data. 

**Table 3:** Antibiogram of Salmonella species

Organisms	Antibiotic name	Number tested	% Resistant ®	%Sensitive (S)	%Or 95% CI	%S 95% CI
Salmonella sp.	Ampicillin	1,192	95.55369	4.446309	94.2-96.6	3.4-5.8
Salmonella sp.	Amoxicillin/Clavulanic acid	1,709	80.16383	19.83616	78.2-82.0	18.0-21.8
Salmonella sp.	Piperacillin/Tazobactam	2,744	43.54956	56.45044	41.7-45.4	54.6-58.3
Salmonella sp.	Cefoperazone	2,458	43.36859	56.63141	41.4-45.4	54.6-58.6
Salmonella sp.	Ceftazidime	2,741	79.38708	20.61292	77.8-80.9	19.1-22.2
Salmonella sp.	Ceftriaxone	1,718	79.74389	20.25611	77.7-81.6	18.4-22.3
Salmonella sp.	Cefotaxime	1,532	86.16188	13.83812	84.3-87.8	12.2-15.7
Salmonella sp.	Cefepime	2,485	80.24145	19.75855	78.6-81.8	18.2-21.4
Salmonella sp.	Cefixime	354	80.22598	19.77401	75.6-84.2	15.8-24.4
Salmonella sp.	Aztreonam	1,225	75.02041	24.97959	72.5-77.4	22.6-27.5
Salmonella sp.	Doripenem	429	0.4662	99.53381	0.1-1.9	98.1-99.9
Salmonella sp.	Ertapenem	528	3.409091	96.5909	2.1-5.4	94.6-97.9
Salmonella sp.	Imipenem	2,202	2.22525	97.77475	1.7-3.0	97.0-98.3
Salmonella sp.	Meropenem	2,575	2.174757	97.82524	1.7-2.8	97.2-98.3
Salmonella sp.	Amikacin	2,071	9.270884	90.72912	8.1-10.6	89.4-91.9
Salmonella sp.	Gentamicin	2,046	18.4262	81.5738	16.8-20.2	79.8-83.2
Salmonella sp.	Ciprofloxacin	2,457	51.56695	48.43305	49.6-53.6	46.4-50.4
Salmonella sp.	Levofloxacin	661	50.5295	49.4705	46.7-54.4	45.6-53.3
Salmonella sp.	Moxifloxacin	589	55.68761	44.31239	51.6-59.7	40.3-48.4
Salmonella sp.	Co-trimoxazole/ Trimethoprim-Sulfamethoxazole	3	66.66667	33.33334	12.5-98.2	1.8-87.5
Salmonella sp.	Fosfomycin	214	6.542056	93.45795	3.8-11.0	89.0-96.2
Salmonella sp.	Colistin	1,479	1.825558	98.17444	1.2-2.7	97.3-98.8
Salmonella sp.	Polymyxin B	1,385	0.505415	99.49458	0.2-1.1	98.9-99.8
Salmonella sp.	Azithromycin	2,325	33.41936	66.58065	31.5-35.4	64.6-68.5
Salmonella sp.	Chloramphenicol	2,449	86.97427	13.02572	85.6-88.3	11.7-14.4
Salmonella sp.	Tigecycline	2,083	2.640422	97.31157	2.0-3.4	96.5-97.9

**Figure 6:**
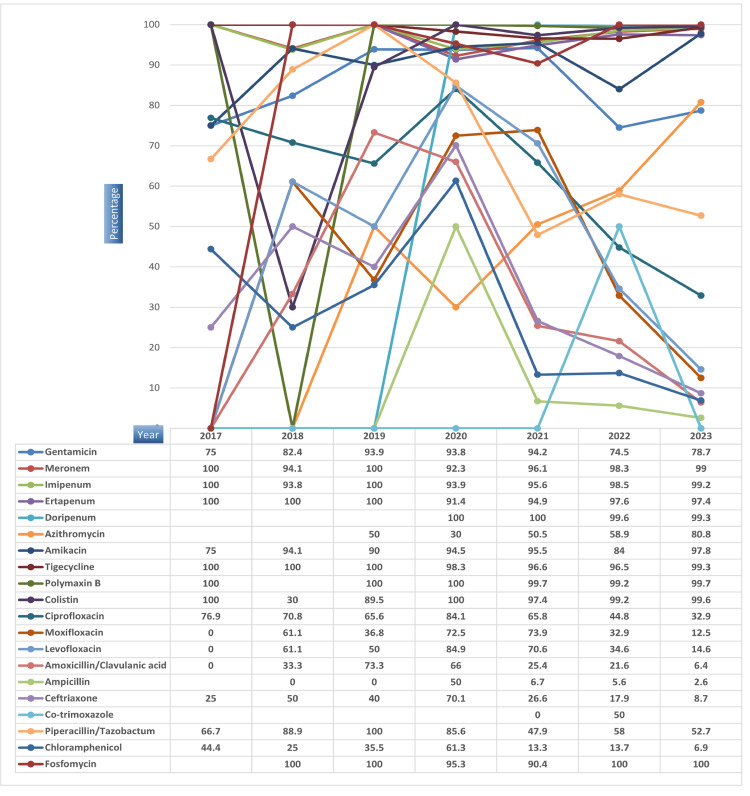
Year-wise susceptibility pattern of Salmonella species from 2017-2023

**Figure 7:**
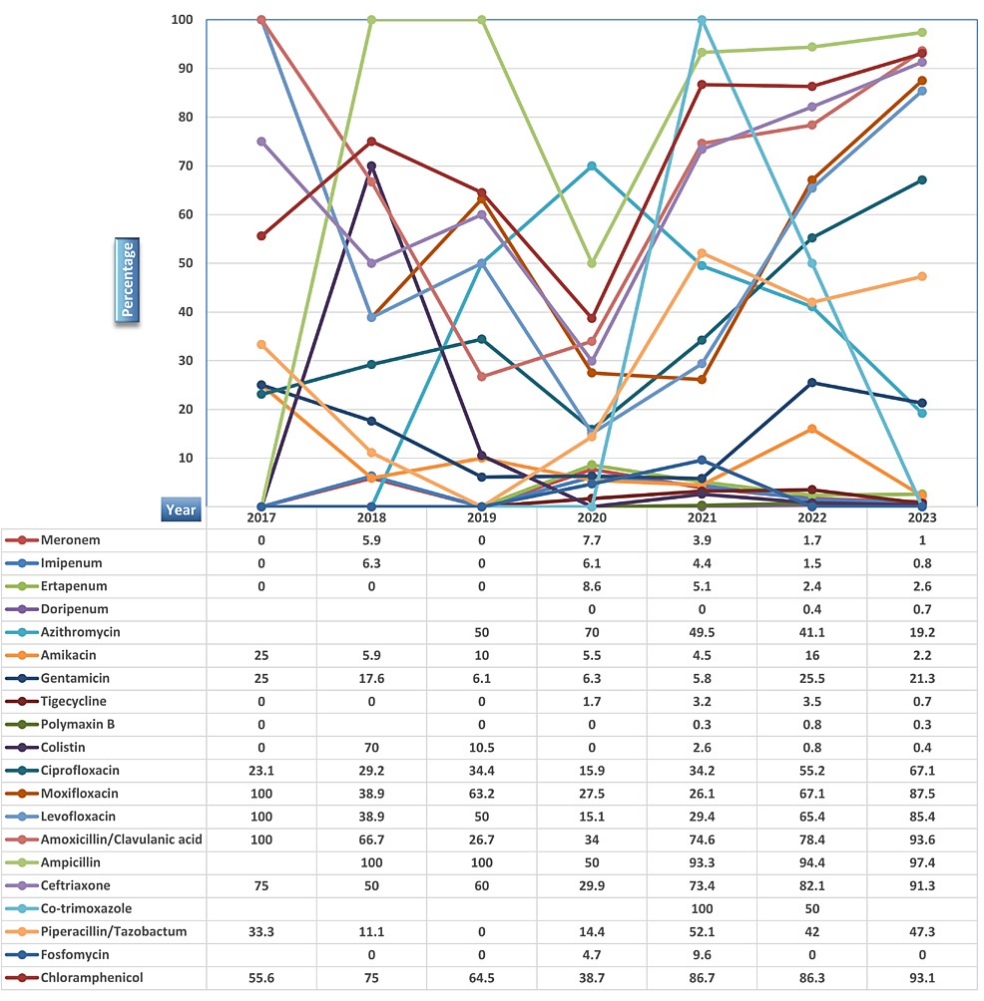
Year-wise antibiotic-resistant pattern of Salmonella species from 2017-2023

The antibiotic susceptibility patterns over the years indicate consistent sensitivity to meropenem, imipenem, ertapenem, doripenem, colistin, tigecycline, and polymyxin B. However, resistance persists for co-trimoxazole/trimethoprim-sulfamethoxazole, chloramphenicol, amoxicillin, ceftriaxone, and fluoroquinolones. Notably, amikacin and fosfomycin exhibit remarkable susceptibility trends, offering promising treatment options. Below is the illustration of the distribution of susceptibility patterns across different years (Figures 6, 7).

## Discussion

Typhoid fever (S. Typhi) and paratyphoid fever (S. Paratyphi) are the causes of enteric fever, an infectious disease that is spread through food and water [9]. Infections that go untreated can cause major morbidity or even death. Asia accounts for the majority of enteric fever cases [1]. The Indian subcontinent is endemic to enteric fever [10]. It is one of Pakistan's biggest health issues. An important issue in the treatment of enteric fever is antibiotic resistance. Pakistan was the site of the greatest S. Typhi outbreak resistant to ceftriaxone in 2017-2018 [11]. To treat enteric fever, understanding the local Salmonella antibiogram and antibiotic stewardship is essential. The purpose of this study was to identify the antibiogram profile, prevalence based on age and gender, and emerging trends of antibiotics in cases of typhoid fever received in KTH and HMC, Peshawar, Pakistan from January 2017 to July 2023.

Males accounted for the majority of cases in the current study. Studies [12-15] also revealed a comparable gender distribution. The fact that men are more likely than women to be exposed to the outdoors and to eat street food in Pakistani society provides a plausible explanation for the preponderance of men [13, 15]. Among the main risk factors for enteric fever is the eating of street food. According to a study done in Karachi, food handlers employed on food streets had a high prevalence of Salmonella carriage [16].

Age distribution analysis shows that cases are most prevalent in the one to 24-year-old age range. Similar age distributions of the cases were described by Sharvani et al. [1]. The same factors that account for male prevalence also increase the likelihood that children and adolescents will come into contact with the disease. In the current study, clustering of instances was also noted in the summer and spring months. In addition, Khan et al. noted a higher frequency in the summer and linked this pattern to the rising use of ice that has been made commercially, chilled beverages, and floods [10]. Similar seasonal trends were documented in India by Sur et al. and Mohanty et al. [14,17].

The majority of Salmonella isolates displayed resistance to primary antibiotics such as amoxicillin, cotrimoxazole, and chloramphenicol, as well as cephalosporins and fluoroquinolones. This aligns with findings by Qamar et al. and Khan et al., who also observed a high prevalence of MDR Salmonella strains with a comparable antibiotic resistance profile in Karachi and Hyderabad [2, 13]. Additionally, Leghari et al. noted lower sensitivity rates for ceftriaxone and cefixime in their study [18]. In contrast, studies conducted in India and Nepal reported lower rates of resistance to first-line antibiotics and a reduced proportion of MDR strains in S. Typhi [1, 19].

In this study, heightened sensitivity was observed for carbapenem, polymyxin B, tigecycline, and colistin. Amikacin exhibited a sensitivity of 90.7%, while gentamicin displayed a consistently high sensitivity of 81.5% over the six years. These antibiotics present promising options for managing typhoid fever, considering their sensitivity profiles and cost-effectiveness. A study in India also noted increased in vitro activity for amikacin and gentamicin [20]. Prolonging the duration of aminoglycoside treatment raises the likelihood of nephrotoxicity, with treatment exceeding 14 days elevating the risk to 50% [21].

Fosfomycin shows a sensitivity of 93.4% and maintained the same sensitivity pattern from 2017-2023. Fosfomycin should be considered a good option for typhoid fever management. A study was conducted in Germany where they used fosfomycin and meropenem for typhoid fever and they were successful [22]. Azithromycin demonstrated an overall sensitivity profile of 66.5%, with an escalating sensitivity pattern from 2017 to 2023. The comparatively lower sensitivity is a cause for concern. Another study on extensively drug-resistant (XDR) S. Typhi in Northern Pakistan indicated sensitivity solely to azithromycin and meropenem [11]. Yousafzai et al.'s investigation into the outbreak of ceftriaxone-resistant S. Typhi in Hyderabad during 2016-2017 reported a similar antibiotic sensitivity profile, specifically highlighting meropenem, imipenem, and azithromycin [12].

Analyzing the annual trends in antibiotic resistance and sensitivity profiles of S. Typhi from 2017 to 2023 reveals a notable rise in resistance to amoxicillin, co-trimoxazole/trimethoprim-sulfamethoxazole, chloramphenicol, third-generation cephalosporins, and fluoroquinolones. This stands in contrast to prior studies conducted in Pakistan, Nepal, and India, where increased sensitivity to first-line drugs, cephalosporins, and fluoroquinolones was reported [5, 8, 12, 13]. This contradicts the findings of our recent study.

In these hospitals (HMC, KTH) we used azithromycin 500 mg once daily for seven days and meropenem 1 gm thrice daily for 10 days but the results of this study suggest that for the empirical treatment of typhoid fever, it is advisable to consider amikacin, gentamycin, fosfomycin, and meropenem. Although aminoglycoside and fosfomycin are not recommended by guidelines clinical trials are needed to check their efficacy for the management of enteric fever. As in Pakistan amikacin is more cost-effective than meropenem and has a good sensitivity, this issue will also be resolved if we find amikacin as an effective drug for enteric fever. In contrast, fluoroquinolones, cephalosporins, co-trimoxazole/trimethoprim-sulfamethoxazole, and azithromycin are not recommended due to their declining sensitivity trends. To address this, continuous antibiogram surveillance is crucial for monitoring antibiotic resistance patterns and guiding empirical treatment decisions. Implementing antimicrobial stewardship programs becomes essential to tackling the escalating issue of antibiotic resistance. Public awareness campaigns should educate individuals about the risk factors and transmission routes of enteric fever, promoting primary prevention measures. Additionally, health authorities may want to contemplate Salmonella vaccination campaigns as a strategic approach to alleviate the disease burden.

## Conclusions

This study highlights a concerning trend of higher prevalence among males, particularly during the summer and spring months. The age group most affected appears to be individuals aged between one and 24 years. Alarmingly, a significant portion of Salmonella isolates exhibit resistance to conventional first-line antibiotics, as well as to ciprofloxacin and third-generation cephalosporins. Notably, azithromycin shows diminished sensitivity compared to amikacin, gentamicin, and fosfomycin.

In light of these findings, urgent action is warranted. Our study suggests the empirical use of amikacin, gentamicin, fosfomycin, and meropenem for the treatment of typhoid fever in Pakistan. However clinical trials are needed to check the efficacy of amikacin, gentamicin, and fosfomycin. To combat the growing threat of antibiotic resistance, it is imperative to implement regular surveillance of Salmonella antibiograms and adopt effective antibiotic stewardship practices. Additionally, public health education initiatives, coupled with Salmonella vaccination programs, are essential for primary disease prevention. By taking these measures, we can address the pressing issue of antibiotic resistance and safeguard public health in our communities.
